# Reflux Extraction Optimization and Antioxidant Activity of Phenolic Compounds from *Pleioblastus amarus* (Keng) Shell

**DOI:** 10.3390/molecules27020362

**Published:** 2022-01-07

**Authors:** Yuan Ma, Ailian Meng, Ping Liu, Yuanyuan Chen, Anqi Yuan, Yemei Dai, Kunyue Ye, Yi Yang, Yiping Wang, Zhuoman Li

**Affiliations:** Key Laboratory of Grain and Oil Processing and Food Safety of Sichuan Province, College of Food and Bioengineering, Xihua University, Chengdu 611743, China; mengailian2021@163.com (A.M.); Liuping20210414@163.com (P.L.); chenyuanyuan0714@163.com (Y.C.); yuananqi980620@163.com (A.Y.); dyemei@163.com (Y.D.); y19140556253@163.com (K.Y.); yang3536317775@163.com (Y.Y.); wyp18782852368@163.com (Y.W.); xm1159447842@163.com (Z.L.)

**Keywords:** *Pleioblastus amarus* (Keng) shell, optimization of extraction, Plackett–Burman experimental design, antioxidant activity

## Abstract

Phenols were extracted from the *Pleioblastus amarus* (Keng) shell (PAS) using ethanol. A Plackett–Burman assessment indicated that the factors affecting polyphenol extraction included the ethanol concentration, extraction temperature, liquid to solid ratio, extraction time, and reflux extraction times; the best extraction parameters were the ethanol concentration of 75%, a 20:1 liquid to solid ratio, and an extraction time of 2.1 h. The number of polyphenols was 7.216 mg/g. Furthermore, the phenol composition analysis showed the presence of p-Coumaric acid (196.88 mg /mL) and rutin (312.9 mg /mL), which were used for the in vitro extraction and determination of the antioxidant activity. According to the A, B, C, and D antioxidant activity assays, the ethyl acetate phase was the strongest with low IC_50_ values of 0.169 ± 0.01 mg/mL, 0.289 ± 0.01 mg/mL, 0.372 ± 0.01 mg/mL, and 1.029 ± 0.03 mg/mL, respectively, confirming high antioxidant activity. For the n-butanol and petroleum ether phases, antioxidant activity was lower. This study showed that the polyphenol extract from *Pleioblastus amarus* (Keng) shell displayed excellent antioxidant activity, enhancing its practical application.

## 1. Introduction

*Pleioblastus amarus* (Keng), also known as bamboo, is widely distributed in China and is high in protein, amino acids, and dietary fiber [1]. Moreover, it is rich in a variety of bioactive substances, including polyphenols, flavonoids, and alkaloids; has anti-inflammatory, antioxidant, antibacterial, and antitumor effects; enhances immune regulation activity, and inhibits tyrosinase [2,3,4]. Naturally abundant phenolic compounds are found in bamboo shells and are generally responsible for functional processes, such as oxidation and aging inhibition and free radical scavenging activity. Although *Pleioblastus amarus* (Keng) shells are typically used as industrial fiber or compost, they are rich in natural flavonoids, polyphenols, cellulose, and polysaccharides. Moreover, many plant-derived phenolic compounds display excellent antioxidant ability. Their preventative role against lipid peroxidation is due to their radical scavenging properties, protecting the cell from ROS formation [5,6] and can be used as a safe, highly stable, and effective natural antioxidants due to their relative nontoxicity and insignificant side effects, which are the main challenges associated with synthetic antioxidants [7]. The functional activities in bamboo leaves and stems have been extensively investigated. Bamboo leaves exhibit antioxidant capacity due to their high polyphenol component [8], and subsequent n-butanol extract have displayed antioxidant activity against the 1,1-diphenyl-2-picrylhydrazyl (DPPH) radical [9]. However, minimal studies are available involving the functional properties of bamboo shoot shells, and the shells are often discarded or used as animal feed. Their utilization has been limited due to the scarcity of data regarding the characterization of their functional components, especially polyphenols [1,10]. Therefore, exploring bamboo shoot extracts can enhance their practical value and improve resource utilization [11].

There are many recent studies reporting that the extraction technique used also affects the results obtained [12,13,14,15]. Extraction is commonly used to ensure the efficient recovery of bioactive compounds from natural sources. This process must be able to extract the bioactive substances from biomass residue while satisfying various other requirements, such as versatility regarding a wide range of compounds and conditions as well as cost efficiency and simple operation [16,17]. Furthermore, the extraction process efficiency can be influenced by different factors. Ethanol is commonly used during the extraction process since it is environmentally friendly and considered safe by the European Food Safety Authority (EFSA) and the FAO/WHO Expert Committee on Food Additives [18]. Phenolic compounds are typically extracted from natural plants using enzyme-assisted, refluxing extraction, microwave-assisted, and ultrasonic-assisted methods [19,20]. Studies have shown that refluxing extraction can increase extraction yield while reducing the extraction time and the amount of solvent used. These advantages over conventional extraction techniques render this method a promising alternative for extracting bioactive natural products [21].

The Plackett–Burman (PB) design is a relatively new tool for screening “key points” in complicated processes and can detect potentially important variables in a complex group of interactions, rendering it suitable for reducing waste generation [22]. Studies have shown that there are many factors that affect the extraction rate of phenols. The apex of the experiment (the maximum response value) found through the PB experiment is the +1 point of the factor, and the −1 point is obtained according to the 1.25 times principle, and the optimal value range of each factor can be determined. On the basis of the PB experiment, the factor with *p* value < 0.05 was selected as the design factor of the response surface experiment, and the Box–Behnken design (BBD) experiment was used to design the response surface to determine the best extraction process of phenols. This study applied the BBD using response surface methodology (RSM). The BBD is one of the most efficient experimental designs since it does not allow combinations for which all factors are simultaneously at their highest or lowest levels. The significant phenolic extraction variables selected in the Plackett–Burman design can improve the detection of optimal regions during sequential optimization using the central composite design and RSM [23].

However, minimal information is available regarding the extraction of polyphenolic antioxidants from the *Pleioblastus amarus* (Keng) shell. In this context, this work deals with the study of the influence of selected operational variables (ratio of solid/liquid, extraction temperature, extraction time, and reflux extraction time) using the combination of the BBD and RSM to optimize the corresponding extraction conditions while obtaining the maximum phenolic compound components [24]. The antioxidant activity was screened with four different methods (DPPH radical scavenging, hydroxyl radical scavenging (•OH), ferric reducing antioxidant power (FRAP), and 2,2′-azino-bis (3-ethylbenzothiazoline-6-sulphonic acid (ABTS)), and the methods were performed to investigate the PAS extractive antioxidant activity in vitro.

## 2. Materials and Methods

### 2.1. Materials 

The *Pleioblastus amarus* (Keng) was purchased in Yibin City, Sichuan Province. Fresh shoot shells were dried, crushed, and passed through a 60-mesh sieve. Gallic acid, rutin, p-Coumaric acid, chlorogenic acid, quercetin, catechin, and resveratrol (all High-Performance Liquid Chromatography (HPLC) grade), ABTS, DPPH, 2,4,6-tri (2-pyridyl)–triazine (TPTZ), and Trolox (≥97%) were purchased from Yuanye Biotechnology Co., Ltd., (Shanghai, China). The methanol (HPLC grade, Licrosolv, 99.9%) and acetonitrile (HPLC grade, Licrosolv, 99.9%) were purchased from Semerfeld Technology Co, Ltd. (Shanghai, China), while all other chemicals (analytical grade) were obtained from the Kelong Chemical Reagent Factory (Chengdu, China).

### 2.2. Sample Preparation

#### Preparation of Extracts from Different Extraction Phases

After optimizing the extraction process, the combined ethanol phase concentrates were collected and extracted three times with equal volumes of ethyl acetate. The extracts were combined, and the ethyl acetate was recovered, and the extracts were freeze-vacuum dried and stored at 4 °C. We took 2 g of freeze-dried concentrated solution, added 10 mL ethyl acetate, petroleum ether, and n-butanol to assist dissolution by ultrasonic to prepare 0.2 g/mL solution.

### 2.3. Methods

#### 2.3.1. Determination of the Total Phenolic Component

The total phenolic component (TPC) was determined using a previously described method [25] with slight modifications. Standard curve production: accurately weighed 0.01 g of gallic acid standard product and placed it in a 10 mL volumetric product, added distilled water to make the volume up to the mark, and obtained a 1.0 mg/mL gallic acid standard product solution, which was marked as mother liquor. Measured 0 mL, 0.1 mL, 0.2 mL, 0.3 mL, 0.4 mL, and 0.5 mL of 1.0 mg/mL standard solution in a 10 mL volumetric flask, and processed them according to the Folin colorimetric method. First added 0.5 mL Folin’s reagent to make the mixture react for 1 min, after which 1.5 mL of a 20% Na_2_CO_3_ solution was added. Ultrapure water was added until reaching a constant volume, and the mixture was reacted at 75 °C for 10 min, after which the absorbance was measured at a wavelength of 760 nm using an ultraviolet–visible absorption spectrometer (7200, Unico (Shanghai) Instrument Co., Ltd., Shanghai, China). According to the measurement result of the standard curve of gallic acid, the regression equation y = 78.607x + 0.0716, R^2^ = 0.9959 was obtained. Sample determination: 0.5 mL of the extract was diluted and followed the previous method. The result is expressed in dry weight of gallic acid equivalent (mg GAE/g DW). The total phenolic content (TPC) of the extract can be expressed as mg chlorogenic acid equivalent per gram of dry weight sample according to the regression equation. The total phenolic component was calculated according to the following Equation (1).

(1) $$X=\frac{\rho \times V\times N}{m}$$

where X represents the polyphenol component in the sample (the *Pleioblastus amarus* (Keng) shell contain milligrams equivalent to gallic acid/mg chlorogenic acid/g). *ρ* denotes the polyphenol mass concentration in the test solution designed according to the standard curve equation/(mg/mL). V is the volume of the test solution/mL. N is the dilution factor, and m is the mass of the sample/g.

#### 2.3.2. Determination of Total Flavonoid Content

The total flavonoid component in the *Pleioblastus amarus* (Keng) shell alcohol extracts were analyzed using the sodium nitrite–aluminum nitrate colorimetry method adapted from a previously delineated technique [25,26], with slight modifications. Rutin (0–120 mg/L) was used as the calibration standard. A 1.0 mL sample of the total flavonoid content (TFC) extract derived from the *Pleioblastus amarus* (Keng) shell, and the ethyl acetate, butanol, petroleum ether extracts were placed in a 10 mL volumetric flask and diluted, after which a 70% ethanol solution was added until the mixture reached the mark on the container. Then, 2 mL of an aluminum chloride solution (0.1 mol/L) and 3 mL of a potassium acetate solution (1.0 mol/L) were added to 1.0 mL of the diluted mixture. After incubation for 30 min in the dark at room temperature, the absorbance was measured at 420 nm. 

#### 2.3.3. HPLC Determination of Reference Polyphenols in *Pleioblastus amarus* (Keng) Shell Extract

With reference to Gao’s [27] research, the standard solution was prepared by accurately weighing 5.0 mg each of catechin, chlorogenic acid, rutin, resveratrol, p-Coumaric acid, quercetin, and gallic acid standards, which were dissolved and diluted to volume with chromatography grade methanol 5 mL. A 1 mg/mL standard stock solution was obtained and diluted into standard solutions of different mass concentrations, filtered through a 0.45 μm organic filtration membrane, and set aside.

The sample solution was prepared by diluting the *Pleioblastus amarus* (Keng) husk polyphenol extract to 1 mL, passing it through a 0.45 μm organic filtration membrane, and set aside.

Chromatographic conditions: Column: Inertsil ODS (C18) (4.6 × 250 mm); Mobile phase conditions: Phase A 0.15% formic acid solution, Phase B 100% acetonitrile; Flow rate: 0.7 mL/min; Injection volume: 20 μL; Column temperature: 35 °C; Detector: Detecting wavelength 280 nm; Gradient elution procedure: 0~5 min, B phase 10%; 5~50 min, B phase 40%; 50~60 min, B phase 10%.

#### 2.3.4. Determination of Antioxidant Activity of Different Extract Phases In Vitro

##### DPPH Radical Scavenging Activity Assay

The DPPH radical scavenging activity of the polysaccharides was determined using a previously reported method with slight modifications [28]. The standard curve was prepared using a 20~160 μmol/L Trolox standard solution, Y = 886.06x + 31.757, R^2^ = 0.9982. Then, 1 mL of the standard solution and 4.5 mL of a 100 μmol/L DPPH methanol solution (7.856 mg DPPH was accurately weighed and diluted to 200 mL with methanol) were thoroughly mixed and placed in the dark at room temperature for 30 min, after which the absorbance was measured at 517 nm (Experiment A). The control group was treated with 4.5 mL anhydrous methanol instead of the DPPH solution (A control), while the blank group was treated with 1 mL distilled water instead of the sample (A blank). The DPPH free radical scavenging rate of the Trolox was calculated using Formula (2).

(2) $$\mathrm{Scavenging}\;\mathrm{activity}\left(\%\right)=\left(A_{\mathrm{blank}}-\left(A_{\mathrm{real}}-A_{\mathrm{control}}\right)\right)/A_{\mathrm{blank}}\times 100$$

##### The •OH Activity Assay

The •OH activity was measured using a previously described method with some modifications [29]. Different sample solution concentrations (0.1 mg/mL~2.0 mg/mL) were mixed with 1 mL of a 9 mmol/L ferric sulfate (FeSO_4_) solution and 1 mL of an 88 mmol/L hydrogen peroxide(H_2_O_2_) solution in test tubes and shaken well for 10 min. Next, 1 mL of a water and salicylic acid solution was added and shaken well, after which the absorbance value (A_1_) was measured at a wavelength of 510 nm. The scavenging hydroxyl radical activity was evaluated employing the following Equation (3).

(3) $$\mathrm{Scavenging}\;\mathrm{activity}\left(\%\right)=\left[1-\left(A_{1}-A_{2}\right)/A_{3}\right]\times 100$$

where A_3_ is the absorbance of the reaction solution without a tested sample. A_1_ denotes the absorbance of the sample, and A_2_ signifies the absorbance of the sample in identical conditions as A_1_ with water instead of a sample solution.

##### The FRAP Assay

The FRAP activity was measured according to a previously reported method with some modifications [30].

To obtain the FeSO_4_ curve, a FeSO_4_ solution was prepared with absolute ethanol (concentration gradient 25 μmol/L~800 μmol/L). Then, 2.7 mL FRAP solution was added to 0.3 mL of each FeSO_4_ solution, shaken well, and allowed to react at 37 °C for 10 min, after which the absorbance was measured at a wavelength of 593 nm. A curve was drawn with concentration (X) as the abscissa and absorbance (Y) as the ordinate, r = 0.9992.

The FeSO_4_ solution was replaced by the extract for sample measurement following the method mentioned above. The FeSO_4_ concentration was proportional to the antioxidant activity of the extract, and the clearance rate was calculated. The results were expressed as μmol Trolox/g DW.

##### ABTS Radical Scavenging Capacity

The ABTS radical scavenging capacity was determined using a previously described method with slight modifications [31]. An ABTS stock solution was prepared by mixing equal volumes of 7 mM ABTS and 2.45 mM potassium persulfate. After 12–16 h at room temperature (25 ± 2 °C), the stock solution was diluted with 70% ethanol to an absorbance of 0.7 (±0.2) and measured at 734 nm using a spectrophotometer. Then, the different sample solutions (0.15 mL, 0.005–2 mg/mL) were mixed with 2 mL of a diluted ABTS solution and reacted for 6 min at room temperature. The absorbance was measured at 734 nm, and ascorbic acid was used as a positive control. The scavenging ability (%) was calculated as following Equation (4).

(4) $$\mathrm{Scavenging}\;\mathrm{ability}\left(\%\right)=\left(1-A_{1}/A_{2}\right)\times 100$$

where A_1_ is the absorbance of the sample reaction solution, and A_2_ is the absorbance of the 70% ethanol.

### 2.4. Experimental Design

#### 2.4.1. Single Factor Experimental Design

The effect of the ethanol concentration, extraction time, extraction temperature, liquid to solid ratio, and refluxing time on the TFA and TPC extraction yield were investigated using a single factor experimental design. Each experimental factor was optimized, while the others were kept constant. Each experiment was performed in triplicate.

When investigating the impact of the solvent-to-raw material ratio in a range of 15:1–25:1, the extraction temperature, extraction time, ethanol concentration, and refluxing time were set to 70 °C, 1 h, 70%, and one time, respectively.

When examining the effect of the extraction temperature in a range of 60 °C to 70 °C, the solvent to raw material ratio, extraction time, ethanol concentration, and refluxing time were set to 20:1, 1 h, 70%, and one time, respectively.

To assess the impact of the extraction time (1–3 h), the solvent to raw material ratio, extraction temperature, ethanol concentration, and refluxing time were set to 20:1, 70 °C, 1 h, 70%, and one time, respectively.

When examining the influence of the ethanol concentration (ranging from 60% to 80%), the solvent to raw material ratio, extraction time, extraction temperature, and refluxing time were set to 20:1, 70 °C, 70%, and one time, respectively. Furthermore, when evaluating the effect of the refluxing time (ranging from one to five), the solvent to raw material ratio, ethanol concentration, extraction time, and extraction temperature were set to 20:1, 70%, 1 h, and 70 °C, respectively.

#### 2.4.2. Plackett–Burman (PB) and Box–Behnken Design (BBD) 

The relevant factors for the polyphenolic extraction and the appropriate range for each factor were preliminarily determined based on the single factor experimental results. RSM was applied to establish the optimum phenolic compound yield from *Pleioblastus amarus* (Keng). A 12 run, nongeometric PB with five factors varied at one level in a combined pattern of the Hamada and Wu matrices (Table 1).

### 2.5. Statistical Analysis

All statistical analyses were conducted using SPSS 24 and Origin 8.5. Data are presented as mean ± standard errors in the Figures. All data obtained in this study were subjected to analysis of variance (ANOVA) followed by Duncan’s multiple range test to determine significant differences among the means at an α = 0.05 level.

## 3. Results

### 3.1. Single Factor Experimental Analysis

**Figure 1:**
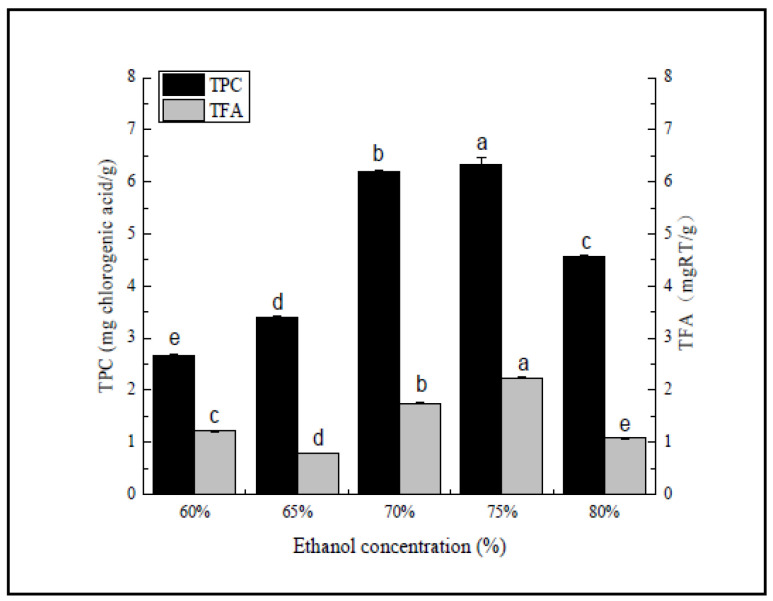
Ethanol concentration (%). Three independent experiments were carried out for each analysis. Result is expressed as a mean ± SD (*n* = 3). Values with the different letters in the column are significantly different by Duncan’s multiple range test (*p* < 0.05).

As shown in Figure 1, the extraction quantity of the polyphenol and total flavonoid component first increased, followed by a decline in conjunction with a higher ethanol volume fraction, reaching a maximum of 75%, consisting of 6.33 mg/chlorogenic acid/g and 2.23 mg RT/g. According to the principle of compatibility, solvents with similar polarity to the bitter bamboo husk polyphenols and total flavonoids allowed them to fully dissolve in the solvent and be extracted. Plant polyphenols often form stable complexes with proteins in the form of hydrogen bonds [32]. The ability of the solvent to destroy hydrogen bonds weakened when the ethanol volume fraction was too low, reducing the extraction rate. Changes in the solvent concentration caused polarity and solubility modifications, resulting in extraction yield changes, as is the case with TPC. These polarity and solubility phenomena could be attributed to the functional groups attached to the compound and its physical and chemical bonding with the solvent molecules [9], modifying the solvent polarity. At a slightly higher ethanol volume fraction, the low solvent polarity decreased the extraction rate and possibly increased the dissolution of fat-soluble substances, such as chlorophyll. Therefore, 70%, 75%, and 80% ethanol were selected as the extraction solvent.

**Figure 2:**
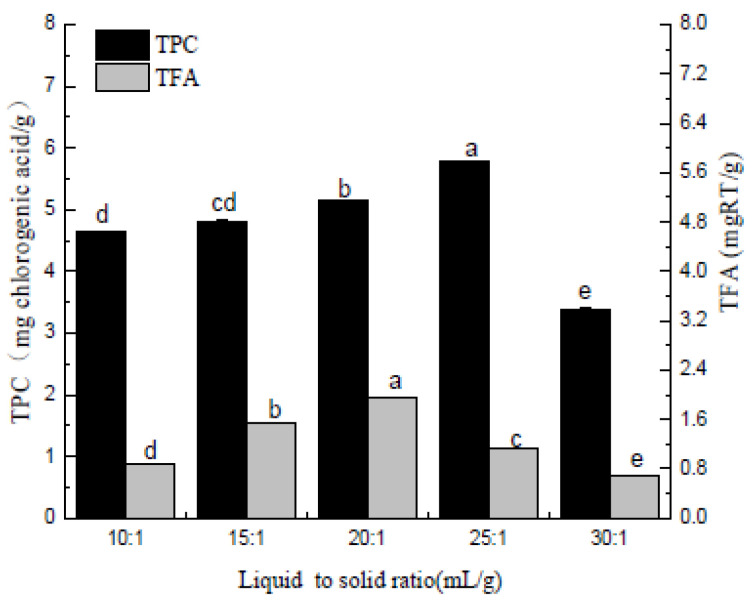
Liquid to solid ratio (mL/g). Three independent experiments were carried out for each analysis. Result is expressed as a mean ± SD (*n* = 3). Values with the different letters in the column are significantly different by Duncan’s multiple range test (*p* < 0.05).

Figure 2 shows the effect of the liquid to solid ratio on the extraction of polyphenols and flavonoids. Flavonoids increase with the increase of the g liquid to solid ratio until it reaches the maximum at 25:1 mL/g and rapidly decreases when the g liquid to solid ratio increases further. The extraction number of polyphenols reaches the maximum when the liquid to solid ratio reaches 20:1 mL/g, and then decreases as the g liquid to solid ratio increases. Taken together, the g liquid to solid ratio is within the experimental range of 10:1~20:1 mL/g, and the extraction number of *Pleioblastus amarus* (Keng) shell polyphenols and total flavonoids changes consistently with the increase of the liquid to solid ratios. The total flavonoid extraction volume displayed a gradual change when the liquid to solid ratios exceeded 20:1 mL/g, which could be attributed to an increase in the amount of solvent that expanded the contact area between the target product and the solvent. However, when the material to liquid ratio reached a specific range, most of the target products in the *Pleioblastus amarus* (Keng) husk were dissolved, decreasing the extraction volume. Therefore, in the subsequent response surface experiment, the three liquid to solid ratios levels were set to 20:1, 25:1, and 30:1 mL/g.

**Figure 3:**
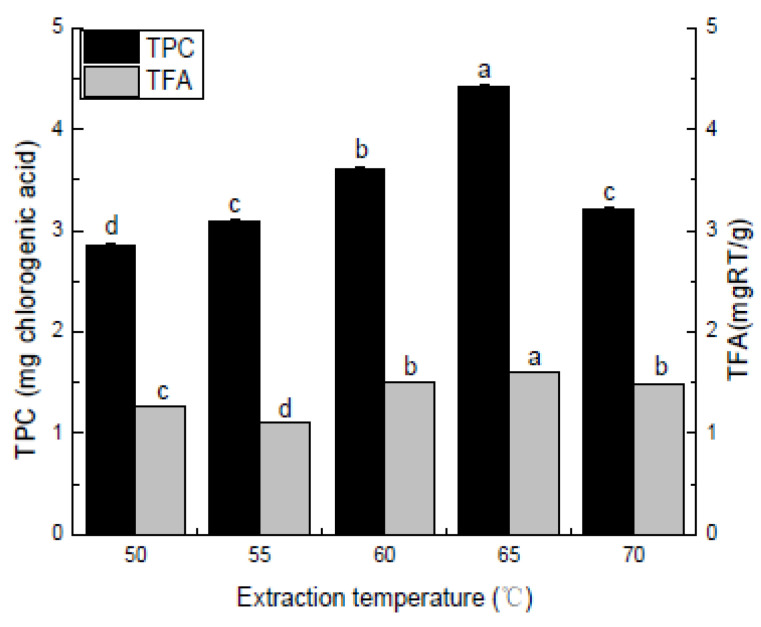
Extraction temperature (°C). Three independent experiments were carried out for each analysis. Result is expressed as a mean ± SD (*n* = 3). Values with the different letters in the column are significantly different by Duncan’s multiple range test (*p* < 0.05).

As shown in Figure 3, the extraction temperatures ranged between 50 °C and 65 °C. The polyphenol and flavonoid components extracted from the *Pleioblastus amarus* (Keng) husk showed an initial increase, followed by a decline in conjunction with a gradual rise in the extraction temperature. The extraction rates of the two active substances gradually increased from 65 °C to 70 °C. The TPC decreased slowly when the temperature exceeded 65 °C. The higher extraction temperature increased the extraction efficiency by raising the solubility of the solute and diffusion coefficients. Heating can soften the plant tissues and disrupt the phenol–protein and phenol–polysaccharide interactions, allowing the extraction of more phenolic compounds [33]. When the temperature exceeded 65 °C, the polyphenol and flavonoid extraction rates showed a decreasing trend. This change can be attributed to the fact that an appropriate increase in temperature can accelerate the penetration, diffusion, and dissolution of flavonoids and polyphenols, making the target product easier to extract. However, when the temperature is too high, it not only promotes flavonoid and polyphenol oxidation but destroys the active substance structure, decreasing the extraction rate. Therefore, the three levels in the climbing experiment were set to 60 °C, 65 °C, and 70 °C.

**Figure 4:**
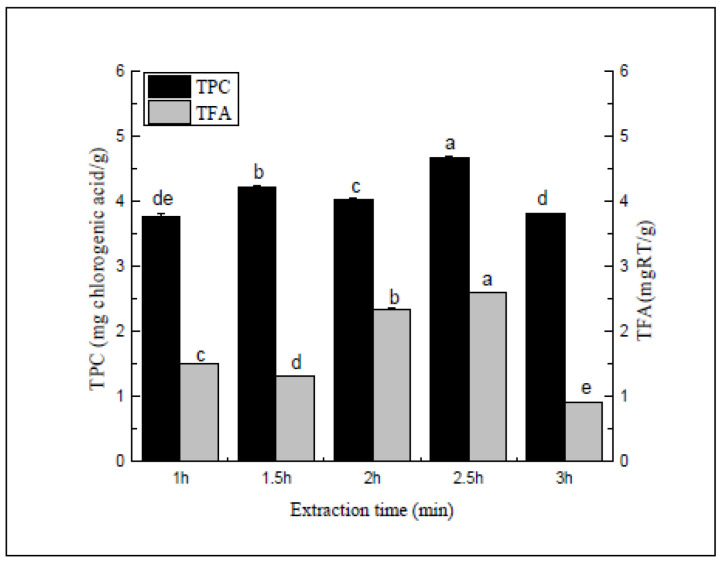
Extraction time (min). Three independent experiments were carried out for each analysis. Result is expressed as a mean ± SD (*n* = 3). Values with the different letters in the column are significantly different by Duncan’s multiple range test (*p* < 0.05).

According to Figure 4, the extraction rate tended to decrease within 1 h to 2 h, while the differences were insignificant. After 2 h to 3 h, the polyphenol and flavonoid extraction rates showed an initial increase, followed by a decrease. As the refluxing time increased, a small number of flavonoids and polyphenols in the sample no longer dissolved in the solvent. However, the extracted samples might be vulnerable to degradation due to an extended extraction time. Therefore, during the climbing experiment, the three extraction times were set to 2 h, 2.5 h, and 3 h.

**Figure 5:**
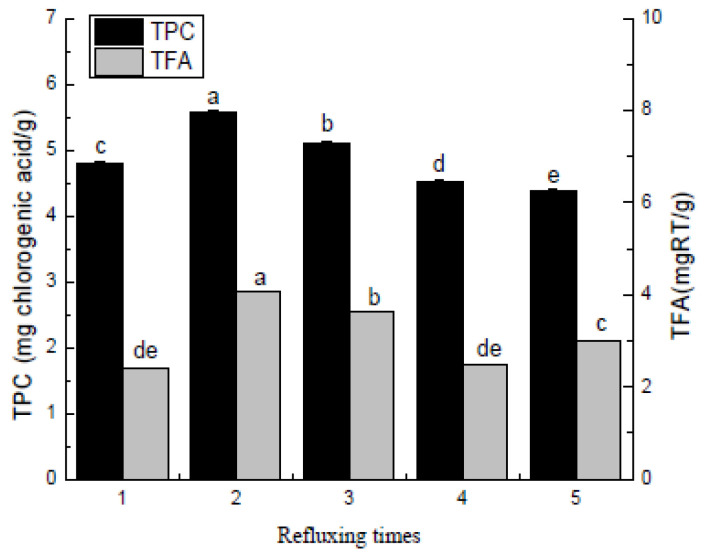
Refluxing times. Three independent experiments were carried out for each analysis. Result is expressed as a mean ± SD (*n* = 3). Values with the different letters in the column are significantly different by Duncan’s multiple range test (*p* < 0.05).

As shown in Figure 5, the impact of the number of refluxing cycles on the extracted polyphenol and flavonoid components from the *Pleioblastus amarus* (Keng) shell was as follows: One to two refluxing cycles gradually increased the polyphenol and flavonoid components, while three refluxing cycles caused a gradual decline. A higher number of refluxing cycles may increase the ethanol solution component, changing the material to liquid ratio and affecting the extraction rate. Furthermore, an excessive material to liquid ratio can also lead to a waste of resources. Therefore, the number of returns was set to one, two, or three cycles. 

### 3.2. PB and BBD Experimental Design and Analysis

#### 3.2.1. RSM Optimization of the Operating Parameters

The PB test with *N* = 12 was used to screen out the significant factors affecting the total phenolic compound extraction rate from the *Pleioblastus amarus* (Keng) shell using five single factors. The PB design matrix and results are shown in Table 2, showing that the material to liquid ratio, extraction temperature, and extraction time represented the significant factors. The variance analysis results are shown in Table 3. The F-test showed that the regression model displayed an exceedingly high F-value (F = 30.78) and an extremely low *p*-value (*p* = 0.0003), indicating that the model was highly significant.

The BBD model was employed to determine the linear and quadratic impact of the variables on the total phenolic compounds. Three independent variables and the corresponding response values obtained from 17 experimental runs are listed in Table 4. In these conditions, the total phenolic compound range was 4.16% to 7.35%. Furthermore, the total phenolic compounds in the *Pleioblastus amarus* (Keng) shell were subjected to RSM optimization. The TPA values obtained via the BBD design and the corresponding predicted values according to the applied second order regression model are shown in Table 5. The statistical test of variance applicable to the second order model is presented in Table 6.

The ANOVA results demonstrated that the liquid to solid ratio (mL/g), ethanol concentration (%), and extraction temperature (°C) were highly significant (*p* < 0.01). Among the items A, B, and C, the quadratic effects and interaction effects of all variables are between A, B, and C. We found that each variable had a significant impact on the production of the TPC. The mathematical mode (Equation (5) that correlates the recovery rate of the TPC with the process variables of the reflux extraction generates the following second order polynomial equation to prove the relationship between the three factors and the response Y.

Y = 7.18 − 0.25 × A − 0.16 × B + 0.26 × C + 0.25 × AB − 0.22 × AC + 0.29 × BC − 1.06 × A^2^ − 1.26 × B^2^ − 1.07 × C^2^ (5)

The regression equation indicates that the material to liquid ratio (A) and extraction time (C) more significantly impacted the number of polyphenols extracted from the *Pleioblastus amarus* (Keng) shell, followed by the ethanol concentration, the liquid to solid ratio, the extraction temperature, and the relationship between the extraction temperature and the extraction time. This interaction had a more substantial effect on the number of polyphenols extracted from the *Pleioblastus amarus* (Keng) shell, while the association between the liquid to solid ratio and the extraction was minimal. Table 6 shows that the F-value of the model was 137.99, *p* < 0.01, indicating that the model reached an extremely significant level. The F-value of the lack of fit term was 0.20, *p* = 0.8913 > 0.05, demonstrating that the difference was not significant, and that the regression model was accurate and reliable. Furthermore, the determination coefficient of the model was (R^2^) = 0.9944, while the calibration determination coefficient was R^2^Adj =0.9872, indicating that the model could explain 98.72% of the response value changes, achieving a high degree of fit. The signal to noise ratio (Adeq precision) was 31.192 > 4.0, showing that the experimental design was reasonable, while the 2.27% coefficient of variance indicated excellent reproducibility. Therefore, the model can be used to determine the process conditions for polyphenol extraction from the *Pleioblastus amarus* (Keng) shell. Regarding the number of polyphenols extracted from the *Pleioblastus amarus* (Keng) shell, the effect of the primary A, B, and C items, as well as the quadratic terms, A^2^, B^2^, and C^2^ were highly significant (*p* < 0.01). The F-value revealed the contribution of the factors in this experiment: extraction time > material to liquid ratio > ethanol concentration.

**Figure 6:**
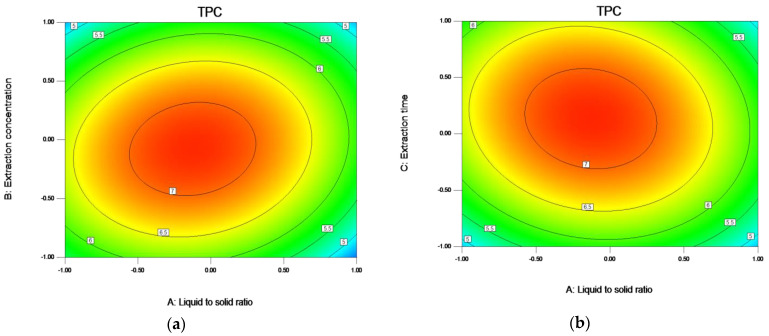

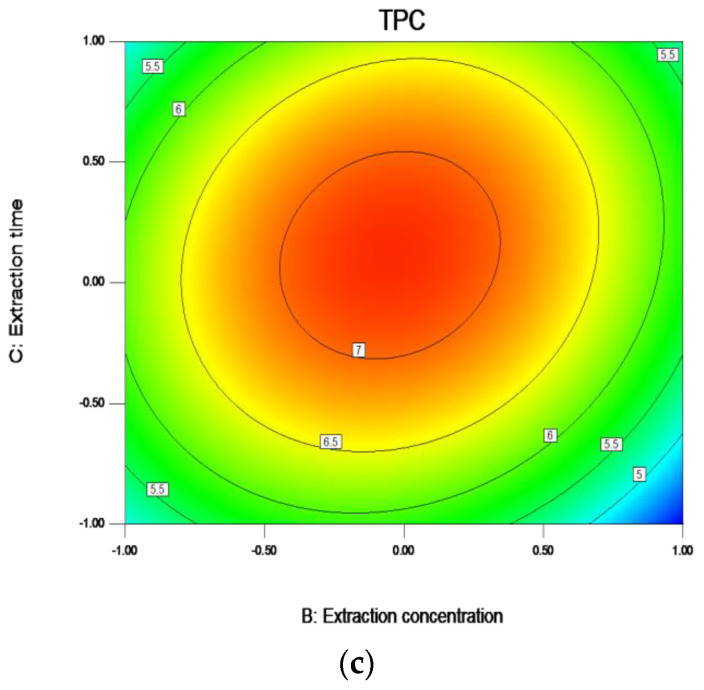
Response contour plots (**a**–**c**) showing liquid to solid ratio (A), ethanol concentration (B), and extraction time (C) on the extraction yield of TPC. Note: The ratio of liquid to solid (A) −1.0, 0.5, 0.0, 0.5, and 1.0, respectively, represent 15:1, 17.5:1, 20:1, 22.5:1, and 25:1; The Ethanol concentration (B) −1.0, 0.5, 0.0, 0.5, and 1.00, respectively, represent 70, 72.5, 75, 77.5, and 80; The Extraction time (C) −1.0, 0.5, 0.0, 0.5, and 1.0, respectively, represent 1.5, 1.75, 2, 2.25, and 2.5.

Figure 6a shows the influence of the interaction between the liquid to solid ratio and the ethanol concentration on the TPC. When the liquid to solid ratio is low, the TPC does not change significantly with the ethanol concentration. When the liquid to solid ratio is 19.32:1, the TPC increased significantly with the change of ethanol concentration.

Figure 6b shows the influence of the interaction between the liquid to solid ratio and extraction time on the TPC. The interaction between the two has a significant effect on the TPC (*p* = 0.0024). When the liquid to solid ratio is 19.32:1, the TPC reaches the maximum when the extraction time is 2.06 h.

Figure 6c shows the influence of the interaction between ethanol concentration and extraction time on the TPC, the interaction between the two has a significant impact on TPC, *p* = 0.0117.

#### 3.2.2. Prediction of the Optimal Response Surface and Verification Testing

The maximum value was used to solve the regression equation using the Design-Expert V8.0.6 software (Stat-Ease Inc., Minneapolis, MN, USA) to obtain the optimal extraction process conditions for the *Pleioblastus amarus* (Keng) husk polyphenols. The optimal combination of factors predicted by the software was a liquid to solid ratio of 19.32:1 mL/g, an ethanol concentration of 74.68%, and an extraction time of 2.06 h. In optimal conditions, the predicted polyphenol extraction number from the *Pleioblastus amarus* (Keng) shell was 7.216 mg/g.

#### 3.2.3. Validation of the Response Surface Test Model

The optimized experimental conditions were refined as follows for an actual situation: a liquid to solid ratios of 20:1 mL/g, an ethanol concentration of 75%, and an extraction time of 2.1 h. Three sets of verification experiments were performed in these conditions. The results are shown in Table 7.

### 3.3. Component Analysis of Polyphenols Derived from Pleioblastus amarus (Keng)

**Figure 7:**
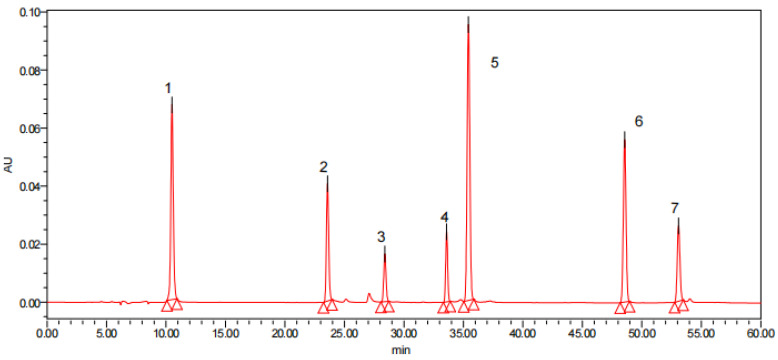
HPLC pictures of 7 polyphenol standards and polyphenol compounds in different parts of *Pleioblastus amarus* (Keng) shell: 1. Gallic acid, 2. Chlorogenic, 3. Catechin, 4. Rutin, 5. P-Coumaric acid, 6. Resveratrol, and 7. Quercetin.

Mixed-sample HPLC plots were generated in the same conditions to obtain the more likely monomeric phenols from the *Pleioblastus amarus* (Keng) shell. To determine the species names of the seven monomeric phenols (Figure 7) for seven polyphenol standards (gallic acid, chlorogenic acid, catechin, p-Coumaric acid, quercetin, rutin, and resveratrol), HPLC plots were used, and Table 8 shows the regression equations for each standard.

The peak time and peak area of the target compound were brought into the standard curve to calculate the content of the target compound in the sample. The experimental results show that the gallic acid, catechin, p-Coumaric acid, quercetin, and rutin component in the *Pleioblastus amarus* (Keng) shell extract were 9.40 mg GAE/100 g, 67.1 mg GAE/100 g, 196.88 mg GAE/100 g, 120.75 mg GAE/100 g, and 312.9 mg GAE/100 g, respectively. The absence of resveratrol and chlorogenic acid may be related to the polyphenol extraction method and growth environment of the *Pleioblastus amarus* (Keng) shell. Li et al [34]. studied the phenolic acids in bamboo shoot shells, and the results showed that the main polyphenols in asparagus shells were p-Coumaric acid, rutin, gallic acid, and catechin.

### 3.4. Polar-Phase Antioxidant Activity Variation in the Pleioblastus amarus (Keng) Phenol Extracts

#### 3.4.1. ABTS Radical Scavenging Capacity

The green oxidation of the ABTS+ free radical fades when coming into contact with an antioxidant. More noticeable fading indicates that the antioxidant capacity of a material is stronger. The research found a significant relationship between antioxidant activity and phenolic components [35].

**Figure 8:**
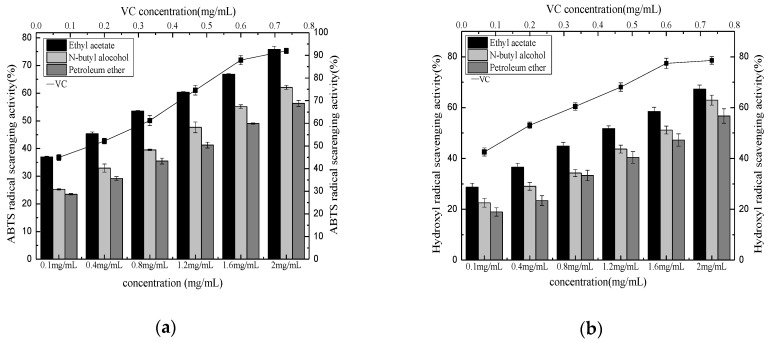
ABTS+ free radical scavenging of different extraction phase (**a**) and hydroxyl radical scavenging activity of different extraction phase (**b**).

Figure 8a shows that the ABTS+ free radical clearance rates by the phenolic compounds and Vitamin VC extracted over three different phases were positively correlated with their mass concentrations. At the same mass concentration, the ABTS+ free radical clearance rate by VC was higher than the phenolic extracts. The ABTS+·scavenging rate reached a maximum at a VC mass concentration of 0.5 mg/mL, followed by a slight change. When the mass concentrations of the phenolic compounds extracted from the *Pleioblastus amarus* (Keng) shell were below 1.6 mg/mL, the ABTS+ free radical scavenging capacity of the petroleum ether and n-butanol extracts displayed no significant differences. However, when this mass concentration level exceeded 1.6 mg/mL, n-butanol exhibited a higher ABTS+ free radical clearance rate than petroleum ether. The relative ABTS+ free radical scavenging rate of ethyl acetate was significantly higher than petroleum ether and n-butanol at the same mass concentration. These differences were substantially reduced at a higher extract concentration. As shown in Table 9, the IC_50_ value indicated that the ABTS+·radical scavenging ability of the VC and phenolic compounds extracted from the *Pleioblastus amarus* (Keng) shell over three different extraction phases exhibited order of VC (IC_50_ mg/mL = 0.150 ± 0.002 ^b^) > VC from the ethyl acetate extraction phase (IC_50_ mg/mL = 0.169 ± 0.001 ^c^) > n-butanol extract VC (IC_50_ mg/mL = 0.350 ± 0.003 ^a^) > petroleum ether extract VC (IC_50_ mg/mL = 0.370 ± 0.002 ^a^).

#### 3.4.2. •OH Radical Scavenging Capacity

•OH represents the most active and toxic reactive oxygen species and can cause oxidative damage to nucleic acids, lipids, proteins, and other macromolecules by interacting with various human molecules. The •OH rate is an indirect indicator during antioxidant activity evaluation.

Figure 8b shows that the clearance rates by the phenolic extracts and VC from the *Pleioblastus amarus* (Keng) shell extracted over three different phases were positively correlated with the mass concentration in general. At the same mass concentration, the hydroxyl radical clearance rate by VC was higher than the phenolic extracts. At a VC concentration of 0.74 mg/mL, the hydroxyl radical clearance rate reached a maximum, while no significant changes were evident. The difference between the •OH ability petroleum ether and n-butanol gradually increased when the phenolic mass concentration exceeded 0.8 mg/mL. The •OH rate of ethyl acetate was significantly higher than petroleum ether and n-butanol at the same mass concentration. These differences gradually decreased at a higher extract concentration. As shown in Table 10, the IC_50_ value indicated that the •OH ability of the phenolic extracts and VC obtained during three different extraction phases displayed the following order: VC (IC_50_ mg/mL = 0.021 ± 0.002 ^d^) > ethyl acetate extraction phase VC (IC_50_ mg/mL = 0.289 ± 0.001 ^c^) > n-butanol extraction phase VC (IC_50_ mg/mL = 0.573 ± 0.002 ^b^) > petroleum ether extraction phase VC (IC_50_ mg/mL = 0.597 ± 0.003 ^a^).

#### 3.4.3. DPPH Radical Scavenging Capacity

DPPH is a stable free radical. Antioxidants can directly pair with lone pair electrons to weaken the DPPH free radical absorption. The antioxidant activity is indirectly evaluated by measuring the degree of absorption reduction. And the DPPH assay is frequently employed to evaluate the antioxidant activity of chemicals in foods due to its high stability, experimental feasibility, and low cost [36,37].

**Figure 9:**
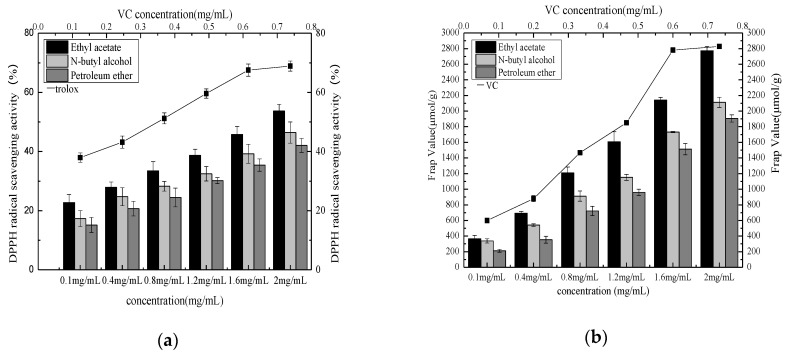
DPPH radical scavenging activity of different extraction phases (**a**) and FRAP value of different extraction phase (**b**).

As shown in Figure 9a and Table 11, the DPPH free radical value of the phenolic extracts from different phases demonstrated a good linear dose–effect relationship within the mass concentration range. At the same mass concentration, the DPPH free radical scavenging rate of the ethyl acetate phase was higher than the n-butanol and petroleum ether phases. The DPPH free radical scavenging rate of the ethyl acetate phase was strongest at a mass concentration of 2 mg/mL, reaching (53.7 ± 2.27%), which was significantly higher than that of the other extracts (P ethyl acetate phase (IC_50_ mg/mL = 0.372 ± 0.002 ^c^) > n-butanol phase (IC_50_ mg/mL = 0.443 ± 0.004 ^b^) > petroleum ether phase (IC_50_ mg/mL = 0.506 ± 0.002 ^a^).

#### 3.4.4. FRAP Capacity

The FRAP method includes the “ferrous reduction capacity experiment” that uses ferrous ions and TPTZ to generate blue–violet complexes to measure the antioxidant capacity of samples in low pH conditions. It is widely used for analyzing the antioxidant capacity of food and health products. The greater the FRAP value, the stronger the antioxidant capacity. Figure 9b and Table 12 show that the FRAP value gradually rose with an increase in the mass concentration of the phenolic extracts derived from *Pleioblastus amarus* (Keng) shell, while those of the different extraction phases were lower than the VC solution. When the extract concentration ranged between 0.1 mg/mL and 2 mg/mL, the FRAP value of the ethyl acetate phase was higher than the n-butanol and petroleum ether phases. At a mass concentration of 2 mg/mL, the FRAP value of the polyphenol extract from the ethyl acetate phase was the strongest, reaching (2772.1 ± 55.72 μmol/g), which was significantly higher than the other phase extracts (P ethyl acetate phase (IC_50_ mg/mL = 1.029 ± 0.003 ^c^) > n-butanol phase (IC_50_ mg/mL = 1.034 ± 0.002 ^b^) > petroleum ether phase (IC_50_ mg/mL = 1.038 ± 0.001 ^a^). 

## 4. Conclusions

Based on single factor polyphenol extraction from *Pleioblastus amarus* (Keng) shell via refluxing, several factors that have a greater impact on the extraction of polyphenols were screened through PB design. In addition, the extraction process of polyphenols was further optimized by response surface methodology combined with the Box–Behnken design. The *Pleioblastus amarus* (Keng) shell polyphenol extraction process was optimized, while the optimal test process conditions were determined as a liquid to solid ratio of 20:1 mL/g, an ethanol concentration of 75%, and an extraction time of 2.1 h. The polyphenol component obtained in these conditions denotes 7.2 mg chlorogenic acid. The predicted values of the optimum extraction parameters are consistent with the experimental values. Five phenolic compounds, including gallic acid, catechin, p-Coumaric acid, quercetin, and rutin are preliminarily identified via HPLC. The results indicate that among the five monomeric phenols, *Pleioblastus amarus* (Keng) shell contains more phenolic compounds, such as p-Coumaric acid, quercetin, and rutin. Moreover, polyphenols of different polarities were extracted with alcohol to determine their in vitro antioxidant activity, revealing that ethyl acetate displays strong •OH ability. At a crude polyphenol extract concentration of 2 mg/mL, the ethyl acetate inhibition rate of DPPH, ABTS, and hydroxyl free radicals reaches maximum values of 53.7% ± 2.27%, 75.78% ± 1.12%, and 67.32% ± 1.57%, respectively. The FRAP value reaches a maximum of 2772.1% ± 55.7%, while the VC FRAP value is close to that of the ethyl acetate phase extract. The scavenging ability of ethyl acetate relative to the DPPH free radicals and ABTS+ and the FRAP value are higher than those of the n-butanol phase. Compared with petroleum ether, ethyl acetate is a medium to strong polar solvent, indicating that the polyphenols in the *Pleioblastus amarus* (Keng) shell are mainly of medium polarity, and the ethyl acetate extraction phase exhibits strong antioxidant activity. Polyphenols and flavonoids have attracted significant attention due to their excellent antioxidative properties, such as rutin, catechin, and quercetin. The antioxidant activity exhibited by the ethyl acetate phase extract is related to the components of the polyphenol-like compounds contained [38,39,40]. *Pleioblastus amarus* (Keng) shell presents a potential source of natural polyphenol-like compounds and may contribute to the development of new antioxidative agents. This study presents a novel strategy for utilizing *Pleioblastus amarus* (Keng) waste as a cheap source of bioactive compounds.

Different forms of phenolic compounds have similar physiological activities and different biological activities. A comprehensive analysis of the phenolic compounds of bitter bamboo shoots will help scientifically develop their potential health benefits. In addition to free phenols, bound phenols have relatively continuous antioxidant effects in the body, and their potential physiological activities are worthy of further investigation.

## Figures and Tables

**Table 1:** The coded values and corresponding actual values of the extraction parameters used in Plackett–Burman design (Ethanol solution).

Variable Code	Variable	Value	
		−1	+1
X1	Ethanol concentration (/%)	60	80
X2	Liquid to solid ratio (mL/g)	10:1	30:1
X3	Extraction temperature (/°C)	50	70
X4	Extraction time (/h)	1	3
X5	Refluxing times	1	5

**Table 2:** Plackett–Burman experimental design matrix and results.

Experiment No.	Solid/ Solvent	Ethanol Concentration	Extraction Time	Number of Reflows	Extraction Temperature	Total Phenolic Compound
		/%	/h		/°C	/mg/g
1	1	1	−1	1	1	4.26 ± 0.007
2	−1	1	1	−1	1	2.77 ± 0.005
3	1	−1	1	1	−1	4.9 ± 0.004
4	−1	1	−1	1	1	3.52 ± 0.006
5	−1	−1	1	−1	1	4.01 ± 0.007
6	−1	−1	−1	1	−1	4.84 ± 0.006
7	1	−1	−1	−1	1	5.87 ± 0.007
8	1	1	−1	−1	−1	5.29 ± 0.006
9	1	1	1	−1	−1	3.15 ± 0.005
10	−1	1	1	1	−1	3.06 ± 0.006
11	1	−1	1	1	1	4.82 ± 0.005
12	−1	−1	−1	−1	−1	4.99 ± 0.006

**Table 3:** Regression coefficients and statistical significance in the fitted regression model based on Plackett–Burman design.

Item	Coefficient Estimate	Sum of Squares	Mean Square	F-Value	*p*-Value	Significant
Model	4.08	8.05	1.61	6.41	0.0213	*
X1	−0.43	2.26	2.26	9.02	0.0239	*
X2	0.56	3.71	3.71	14.78	0.0085	**
X3	−8.333 × 10^−0.04^	8.33 × 10^−0.06^	8.33 × 10^−0.06^	3.321 × 10^−0.05^	0.9956	
X4	−0.78	1.83	1.83	7.31	0.0354	*
X5	−0.14	0.24	0.24	0.97	0.3624	

Note: *. The difference is significant, *p* < 0.05. **. The difference is significant, *p* < 0.01.

**Table 4:** Independent variables and their levels used for BBD.

Factors	Coded Symbols	Levels		
		−1	0	1
Liquid to solid ratio (mL/g)	A	20:1	25:1	30:1
Ethanol concentration (/%)	B	70	75	80
Extraction time (/h)	C	1.5	2	2.5

**Table 5:** Box–Behnken design (BBD) with the observed responses for yield of total phenolic.

Experiment No.	Liquid to Solid Ratio	Extraction Concentration		Extraction Time	Total Phenolic Compound
	/mL/g	/%		/h	/mg/g
1	20:1	80		2.5	5.30
2	25:1	75		1.5	4.80
3	20:1	80		1.5	4.16
4	20:1	75		2	7.22
5	20:1	70		1.5	5.01
6	15:1	70		2	5.58
7	20:1	75		2	7.01
8	25:1	80		2	4.65
9	20:1	75		2	7.02
10	20:1	70		2.5	4.96
11	20:1	75		2	7.35
12	15:1	75		2.5	5.74
13	25:1	70		2	4.55
14	25:1	75		2.5	4.86
15	15:1	80		2	4.68
16	15:1	75		1.5	4.83
17	20:1	75		2	7.30
X5	−0.14	0.24	0.24	0.97	0.3624

**Table 6:** Analysis of variance (ANOVA) for the experimental results obtained by Box–Behken response surface design.

Source	Sum of Squares	Df	Mean Square	F-Value	*p*-Value	Significant
Model	20.05	9	2.23	137.99	<0.0001	**
A Liquid to solid ratio	0.48	1	0.48	29.94	0.0009	**
B Ethanol concentration	0.22	1	0.22	13.49	0.0079	**
C Extraction temperature	0.53	1	0.53	32.52	0.0007	**
AB	0.25	1	0.25	15.76	0.0054	**
AC	0.19	1	0.19	11.46	0.0117	**
BC	0.34	1	0.34	21.29	0.0024	**
A^2^	4.70	1	4.70	290.84	<0.0001	**
B^2^	6.68	1	6.68	413.84	<0.0001	**
C^2^	4.78	1	4.78	296.20	<0.0001	**
Error	0.11	7	0.016			
Lack of fit	0.015	3	0.0049	0.20	0.8913	Not Significant
Pur error	0.098	4	0.025			
Cor total	20.17	16				
R^2^	0.9944	R^2^Adj	0.9872			

Note: *. The difference is significant, *p* < 0.05. **. The difference is significant, *p* < 0.01.

**Table 7:** Repeat verification experiment results.

No.	Total Phenolic Compounds	Relative Error
1	7.184	0.15%
2	7.208	0.13%
3	7.204	0.16%

**Table 8:** HPLC profile information of 7 phenolic substances.

Proof Sample	Standard Curve	Correlation Coefficient /(R^2^)	Range of Linearity/(g/L)	Retention Time /min
Gallic acid	y = 5 × 10^7^x + 639,963	0.9991	0.05~1.0	10.505
Chlorogenic acid	y = 6 × 10^6^x + 87,892	0.9987	0.05~1.0	23.581
Catechin	y = 9 × 10^6^x – 101,397	0.9987	0.05~0.5	28.400
p-Coumaric acid	y = 8 × 10^7^x + 85,175	0.9992	0.05~0.5	33.420
qQuercetin	y = 3 × 10^7^x – 169,274	0.9999	0.05~1.0	53.087
Rutin	y = 1 × 10^7^x − 175,176	0.9994	0.05~0.5	33.594
Resveratrol	y = 1 × 10^7^x + 141,003	0.9995	0.05~1.0	48.559

**Table 9:** Scavenging effect of *Pleioblastus amarus* (Keng) shell with different extraction phases on ABTS+ free radical.

Extract Phases	Equation of Linear Regression	R^2^	IC50
Ethyl acetate	y = 19.595x + 36.543	R² = 0.9939	0.169 ± 0.001 ^c^
N-butyl alcohol	y = 19.132x + 24.327	R² = 0.9976	0.350 ± 0.003 ^a^
Petroleum ether	y = 16.99x + 21.86	R² = 0.998	0.370 ± 0.002 ^a^
VC	y = 26.378x + 42.007	R² = 0.9858	0.150 ± 0.002 ^b^

Three independent experiments were carried out for each analysis. Result is expressed as a mean ± SD (*n* = 3). Values with the different letters in the column are significantly different by Duncan’s multiple range test (*p* < 0.05).

**Table 10:** Hydroxyl radical scavenging activity of *Pleioblastus amarus* (Keng) shell with different extraction phases on hydroxyl radical.

Extract Phases	Equation of Linear Regression	R^2^	IC_50_
Ethyl acetate	y = 19.644x + 27.979	R² = 0.9956	0.289 ± 0.001 ^c^
N-butyl alcohol	y = 20.618x + 19.66	R² = 0.9889	0.573 ± 0.002 ^b^
Petroleum ether	y = 19.823x + 16.531	R² = 0.9966	0.597 ± 0.003 ^a^
VC	y = 21.834x + 42.328	R² = 0.9931	0.021 ± 0.002 ^d^

Three independent experiments were carried out for each analysis. Result is expressed as a mean ± SD (*n* = 3). Values with the different letters in the column are significantly different by Duncan’s multiple range test (*p* < 0.05).

**Table 11:** Scavenging effect of *Pleioblastus amarus* (Keng) shell with different extraction phases on DPPH free radical.

Extract Phases	Equation of Linear Regression	R_2_	IC50
Ethyl acetate	y = 15.829x + 20.968	R² = 0.9948	0.372 ± 0.002 ^c^
N-butyl alcohol	y = 14.287x + 16.89	R² = 0.9832	0.443 ± 0.004 ^b^
Petroleum ether	y = 13.644x + 14.135	R² = 0.9948	0.506 ± 0.002 ^a^
Trolox	y = 21.241x + 34.813	R² = 0.9949	0.219 ± 0.003 ^d^

Three independent experiments were carried out for each analysis. Result is expressed as a mean ± SD (*n* = 3). Values with the different letters in the column are significantly different by Duncan’s multiple range test (*p* < 0.05).

**Table 12:** FRAP values of *Pleioblastus amarus* (Keng) shell with different extraction phases.

Extract Phases	Equation of Linear Regression	R^2^	IC50
Ethyl acetate	y = 942.09x + 173.71	R² = 0.9856	1.029 ± 0.003 ^c^
N-butyl alcohol	y =1243.5x + 200.79	R² = 0.9954	1.034 ± 0.002 ^b^
Petroleum ether	y = 906.61x + 22.764	R² = 0.9816	1.038 ± 0.001 ^a^
VC	y = 1277.6x + 435.3	R² = 0.9688	0.937 ± 0.002 ^d^

Three independent experiments were carried out for each analysis. Result is expressed as a mean ± SD (*n* = 3). Values with the different letters in the column are significantly different by Duncan’s multiple range test (*p* < 0.05).

## Data Availability

Not applicable.

## Sample Availability

Samples of the compounds are available from the authors.
